# Retraction: Preventive effect of lemon seed flavonoids on carbon tetrachloride-induced liver injury in mice

**DOI:** 10.1039/d3ra90096g

**Published:** 2023-09-27

**Authors:** Ming Yang, Fengjun Sun, Yue Zhou, Mei He, Pu Yao, Yuan Peng, Fei Luo, Fu Liu

**Affiliations:** a Department of Pharmacy, Affiliated Hospital of North Sichuan Medical College Nanchong 637000 Sichuan China ncliufu91@163.com; b Department of Pharmacy, Southwest Hospital, Third Military Medical University (Army Medical University) Chongqing 400038 China

## Abstract

Retraction of ‘Preventive effect of lemon seed flavonoids on carbon tetrachloride-induced liver injury in mice’ by Ming Yang *et al.*, *RSC Adv.*, 2020, **10**, 12800–12809, https://doi.org/10.1039/D0RA01415J.

The Royal Society of Chemistry hereby wholly retracts this *RSC Advances* article due to significant portions of text overlap and due to concerns with the reliability of the data.

There is text overlap with ref. 1 and 2 below, which were not cited.

The raw data provided by the authors for the western blots in Fig. 2, 3 and 4 shows evidence of manipulation. The data has no background and after image analysis hidden fragments became visible.

Given the significance of the concerns about the validity of both the data in the article and the raw data provided by the authors, the findings presented in this paper are not reliable.

Ming Yang, Fengjun Sun, Yue Zhou, Mei He, Yuan Pen and Fei Luo do not agree to the retraction. The other authors were informed about the retraction but did not respond.

Signed: Laura Fisher, Executive Editor, *RSC Advances*

Date: 20th September 2023

## Supplementary Material
